# An Optimized Corn-Based Artificial Diet Reshapes the Larval Metabolome, Leading to Mixed Improvements in Longevity and Reproductive Traits in *Spodoptera litura*

**DOI:** 10.3390/insects17070725

**Published:** 2026-07-13

**Authors:** Aning Fan, Nipapan Kanjana, Xiaotong Xu, Yuanfei Li, Hanqi Li, Yuyan Li, Jianjun Mao, Junjie Zhang, Lisheng Zhang

**Affiliations:** 1 Jilin Province Technology Research Center of Biological Control Engineering, Institute of Biological Control, Jilin Agricultural University, Changchun 130118, China; fananing@126.com (A.F.);; 2 State Key Laboratory for Biology of Plant Diseases and Insect Pests, Institute of Plant Protection, Chinese Academy of Agricultural Sciences, Beijing 100193, China; 3 The National Engineering Laboratory of Crop Stress Resistance, School of Life Sciences, Anhui Agricultural University, Hefei 230036, China; kangjana1993@gmail.com; 4 Program of Agriculture, Faculty of Agricultural Production, Maejo University, Chiang Mai 50290, Thailand; 5 College of Plant Protection, Shenyang Agricultural University, Shenyang 110866, China; 6 Key Laboratory of Animal Biosafety Risk Prevention and Control (North), Ministry of Agriculture and Rural Affairs, Shanghai Veterinary Research Institute, Chinese Academy of Agricultural Sciences, Shanghai 200241, China

**Keywords:** *Spodoptera litura*, mass rearing, metabolomics, fatty acids, linoleic acid

## Abstract

*Spodoptera litura* (Fabricius) is a major agricultural pest, and the establishment of efficient mass-rearing systems is critical for advancing biological control and integrated pest management strategies. In our previous work, evaluation of 17 artificial diets identified an optimised corn-based formulation (F15) that significantly improved larval performance relative to a standard control (CK) diet. However, the metabolic mechanisms underlying these performance differences remain largely unresolved. Here, we employed untargeted metabolomics based on liquid chromatography–tandem mass spectrometry (LC–MS/MS) to elucidate the biochemical pathways associated with enhanced development in *S. litura* larvae reared on F15 compared with CK. Larvae fed the F15 diet exhibited a prolonged developmental period (21.49 ± 0.86 vs. 20.13 ± 0.53 days), higher emergence rates (1.20 ± 0.27% vs. 0.85 ± 0.10%), and increased body mass during both early (3.28 ± 0.12 vs. 2.93 ± 0.08 mg) and later instars (77.55 ± 2.30 vs. 73.51 ± 1.17 mg) relative to those reared on the control diet. Metabolomic profiling revealed that the CK diet, characterised by lower levels of the essential fatty acid linoleic acid (LA), was associated with reduced metabolite abundance and weaker enrichment of energy-related metabolic pathways. In contrast, the F15 diet contained substantially higher levels of linoleic acid, which is known to contribute to membrane stability and mitochondrial function. Notably, linoleic acid was consistently enriched in the gut of F15-fed larvae, suggesting a correlative central role in promoting metabolic efficiency, growth, and developmental performance. Collectively, these findings provide mechanistic insight into how dietary composition modulates insect metabolism and highlight the nutritional and physiological advantages of corn-based formulations for large-scale rearing. This study offers a foundational theoretical foundation for optimising artificial diets and supports their application in sustainable pest-management programs.

## Simple Summary

*Spodoptera litura* (Fabricius) is an important agricultural pest that causes serious damage to various crops. Efficient and large-scale rearing of this pest is essential for the research and application of biological control and integrated pest management. In previous studies, we tested 17 artificial diets and selected an optimised corn-based diet (F15), which significantly promoted the growth and development of *S. litura* larvae compared with the conventional control diet (CK). However, the internal metabolic mechanisms responsible for these beneficial effects have not been clarified. In this study, we used LC-MS/MS-based untargeted metabolomics to analyse the metabolic differences of *S. litura* larvae fed on F15 and CK diets. The results showed that larvae reared on the F15 diet had higher emergence rates and heavier body mass at both early and late instars. Further analysis revealed that the CK diet was relatively deficient in linoleic acid, an essential fatty acid, leading to lower overall metabolite levels and weaker activation of energy-related metabolic pathways. In contrast, the F15 diet was rich in linoleic acid, which was stably enriched in larval guts and was associated with improved metabolic efficiency, growth, and developmental robustness. These findings reveal the correlative regulatory effects of dietary components on the metabolic physiology of *S. litura*, and clarify the nutritional and physiological advantages of the corn-based optimised diet. This study provides a preliminary reliable theoretical basis for the further optimisation of artificial diets and supports the construction of efficient mass-rearing systems for sustainable pest management.

## 1. Introduction

*Spodoptera litura* (Hübner), commonly known as the tobacco or common cutworm, is a highly polyphagous and destructive lepidopteran pest that feeds on more than 389 plant species worldwide, including cotton, rice, maize, soybean, groundnut, and vegetables [1]. This pest is capable of producing multiple generations per growing season and tolerates high temperatures and humidity, contributing to its rapid spread and economic impact [2]. Effective laboratory studies and pest management strategies rely on the mass-rearing of healthy insects, which requires careful consideration of diet quality and composition [3,4]. Essential dietary components—such as proteins, lipids, minerals, and vitamins play critical roles in driving larval growth, development, and reproductive performance. Accordingly, artificial diets incorporating wheat germ, chickpea powder, soybean, maize, and tomato paste, supplemented with vitamin mixtures, have been widely used for rearing *S. litura* and other noctuid species [5,6].

Among these nutrients, lipids and fatty acids are particularly important because they serve as structural elements of cell membranes, primary energy reserves, and precursors for pheromones and defensive metabolites [7]. Food lipids not only provide energy, fat-soluble vitamins, and essential fatty acids, but also play a significant role in shaping the sensory characteristics of the food [8] The biosynthesis of linoleic acid (LIN; 18:2n-6), generally considered an essential dietary nutrient for animals [9]. Conjugated linoleic acid demonstrated desirable insecticidal properties, including increased larval mortality, slowed larval development, antifeedant effects, and decreased egg viability after maternal ingestion [10].

Various approaches have been developed for insect rearing. For example, *Cnaphalocrocis medinalis* can be maintained on natural, artificial, or mixed diets, while *S. frugiperda* has been successfully reared on protein- and lipid-enriched artificial diets with continuous refinement [11]. In mass-rearing programs for biological control, diet quality strongly influences the performance of parasitoids such as *Telenomus remus*, which maintain stable parasitism rates when reared on *S. litura* eggs [12,13]. In addition, modern pest management increasingly integrates environmentally friendly strategies, such as the release of irradiated sterile males to suppress wild populations of *S. litura* [14,15].

Despite the recognised importance of diet composition, systematic characterisation of how different artificial diets shape the metabolic landscape of *S. litura* remains limited. A metabolomics-based understanding is essential, as metabolism directly links nutrient intake to growth, reproduction, stress resistance, and overall fitness.

In this study, untargeted metabolomics based on high-performance liquid chromatography coupled with mass spectrometry (LC-MS) was employed to characterise gut metabolite profiles of *S. litura* larvae reared on either a standard control diet (CK) or an optimised corn-based formulation (F15). Integrating the metabolomic dataset with life-history trait measurements encompassing larval development, adult longevity, oviposition parameters, and emergence rate, we identified linoleic acid and a pyrimidine metabolite as key discriminatory compounds whose abundance was positively associated with alterations in developmental progression and enhanced physiological performance in F15-fed larvae. It is important to emphasise, however, that the associations reported here are correlational in nature; causal relationships between specific metabolites and phenotypic improvements remain to be established through direct supplementation experiments and functional genetic analyses. Notwithstanding this limitation, the present findings provide mechanistic insight into how dietary lipid composition influences insect physiological performance and offer a systematic metabolomic reference for the refinement of artificial diets used in mass-rearing and pest management programmes. Although the beneficial effects of essential fatty acids on lepidopteran growth are well established, the present study contributes a species-specific, condition-controlled metabolomic comparison between a practical corn-based diet and a conventional artificial diet, providing a quantitative framework that can directly inform diet optimisation strategies for *S. litura* rearing at scale.

## 2. Materials and Methods

### 2.1. Insect Rearing

A laboratory colony of *Spodoptera litura* was established from individuals obtained from the Experimental Base of the Institute of Plant Protection, Chinese Academy of Agricultural Sciences (Langfang, Hebei, China; 39°32′18″ N, 116°41′01″ E). The colony was maintained at 26 ± 1 °C, 70 ± 5% relative humidity, and a 16:8 h light: dark photoperiod. To minimise cannibalism, larvae were individually reared in glass tubes (7.5 cm × 2.5 cm). First-instar larvae (<24 h old) were used for all experiments. Adults were kept in plastic containers (6.5 cm × 12 cm) lined with wax paper to provide oviposition surfaces and were supplied daily with a 20% (*v*/*v*) honey solution absorbed on cotton.

For metabolomic analysis, sixth-instar larvae from each diet group were collected and immediately processed for LC–MS/MS to characterise gut metabolite composition.

### 2.2. Artificial Diet Formulation and Assessment of Life-History Traits

A total of 18 experimental diets were formulated by adjusting the proportions of corn flour, soybean powder, yeast, and wheat bran, as detailed in Table 1. Diet CK served as the standard control, and diet F15 was selected for subsequent metabolomic comparison.

Newly laid egg masses were placed in 100 mm Petri dishes lined with moist filter paper and maintained under controlled environmental conditions. Egg hatching was monitored twice daily.

Upon adult emergence, individuals from each diet group were observed daily to record total fecundity, oviposition period, pre-oviposition period, egg-hatch rate, and adult lifespan. Additional parameters included larval emergence rate and sex ratio. Eggs laid on wax paper or mesh were transferred to Petri dishes and sealed with Parafilm, with aeration holes added using insect pins.

### 2.3. Processing of RAW Metabolomic Data

**Figure 1:**
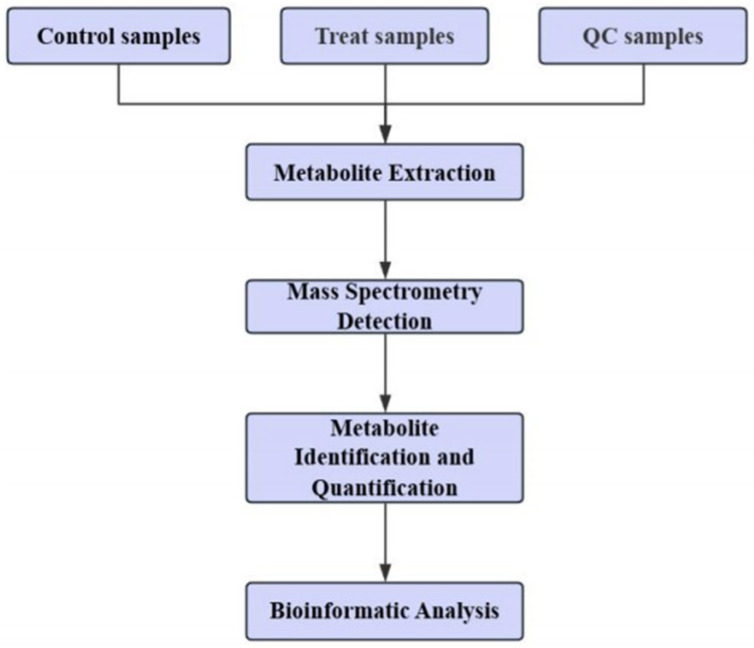
Schematic workflow of the metabolomic analysis of *S. litura*. Samples were collected, metabolites extracted, QC samples prepared, and all extracts analysed by mass spectrometry, followed by data processing and statistical analysis.

RAW metabolomic data were processed using dedicated data-processing software to perform peak alignment, retention-time correction, and peak-area extraction. The resulting feature matrix was then subjected to metabolite identification and quantification, followed by data pre-processing steps (including filtering, normalisation, and missing-value imputation). After ensuring overall data quality through QC-based assessments, the dataset was used for downstream statistical analysis and visualisation. The main workflow consisted of sample collection, metabolite extraction, QC preparation, mass spectrometry acquisition, and data analysis, as illustrated in Figure 1.

#### Experimental Quality Control

Quality control was evaluated using the total ion chromatograms (TICs) of QC samples. The high degree of spectral overlap indicates that both retention times and peak intensities were highly consistent across injections, demonstrating minimal instrument-driven variation throughout the experiment [16]. To ensure a high-quality dataset, the relative standard deviation (RSD) of characteristic peaks in QC samples was required to remain below 30%. Peaks exceeding this threshold were removed during Quality Assurance (QA) to eliminate features with poor reproducibility. A lower RSD in QC ion-peak intensities reflects greater analytical stability and is considered a key indicator of overall data quality [17].

### 2.4. Metabolite Identification and Analysis

Accurate metabolite identification is challenging because many metabolites share similar isomers and molecular masses, making it difficult to distinguish their structures with high confidence. The choice of identification method directly influences the reliability of metabolite annotation and, consequently, the robustness of downstream analyses and functional interpretation. To address this issue, leading metabolomics experts, including Oliver Fiehn, recommend that metabolome profiling studies explicitly report the confidence rank of each identified metabolite [18]. Notably, several recent Cell publications follow this recommendation by applying the Metabolomics Standards Initiative (MSI) criteria and clearly defining the MSI identification ranks within their Methods sections [19,20]. The requirement for high-confidence identification has become increasingly emphasised by reviewers, and metabolite lists lacking confirmation through standard-spectrum matching are frequently questioned or rejected. International guidelines on identification levels were first formalised in 2007 by the MSI Chemical Analysis Working Group (MSI: http://MSI-Workgroups.sourceforge.net, accessed on 2 December 2025) [21]. According to these standards, metabolite identification is supported by matching experimental MS/MS spectra, collision energies, and additional structural information against both local databases and public repositories such as the Human Metabolome Database (HMDB; http://www.hmdb.ca, accessed on 2 December 2025), METLIN (http://metlin.scripps.edu, accessed on 2 December 2025), Mass Bank (http://www.massbank.jp, accessed on 2 December 2025) and mzCloud (https://www.mzcloud.org, accessed on 2 December 2025). All putative identifications undergo strict manual inspection of secondary fragmentation spectra to ensure structural accuracy, and only metabolites achieving MSI Level 2 or above are accepted. Metabolomic data were analysed using Metabo Analyst 6.0 for visualisation and multivariate statistical analysis of the final chemical concentration dataset obtained from our compound collection experiments. Data were auto-scaled and log-transformed to approximate a normal distribution. Hierarchical clustering was performed using Euclidean distance and the Ward linkage method.

LC–MS data, including m/z values and retention times, were exported using Profiling Solution^®^ software (version 1.1 Build 104; Shimadzu, Kyoto, Japan). Sample LC–MS fingerprints were processed with Profiling Solution 1.1 Build 104 for mass signal extraction and alignment over 0–12 min and *m*/*z* values of 200–600 Da. The following parameters were applied: ion *m*/*z* tolerance of 25 mDa, ion retention time tolerance of 1.5 min, ion intensity threshold of 1000 counts, 20% isomer valley detection, and inclusion of ions lacking isotope peaks.

In this study, all identified metabolites were further categorised according to their Chemical Taxonomy classes. The proportional distribution of each superclass was visualised using a pie chart, selecting the broadest taxonomy level to provide a clear and interpretable overview.

### 2.5. Statistical Analyses

Principal component analysis (PCA) was conducted using SIMCA-P (version 13.0.0.0), which applies soft independent modelling of class analogies (SIMCA) for multivariate data analysis. Statistical comparisons were performed using Student’s *t*-test, and results are presented as mean ± SEM, with asterisks indicating significant differences relative to control (* *p* < 0.05; ** *p* < 0.01, *** *p* < 0.001). Differential metabolites were annotated and subjected to enrichment analysis using the KEGG (Kyoto Encyclopaedia of Genes and Genomes) database [22].

## 3. Results

### 3.1. Development, Survival, and Reproductive Performance of Spodoptera litura

**Figure 2:**
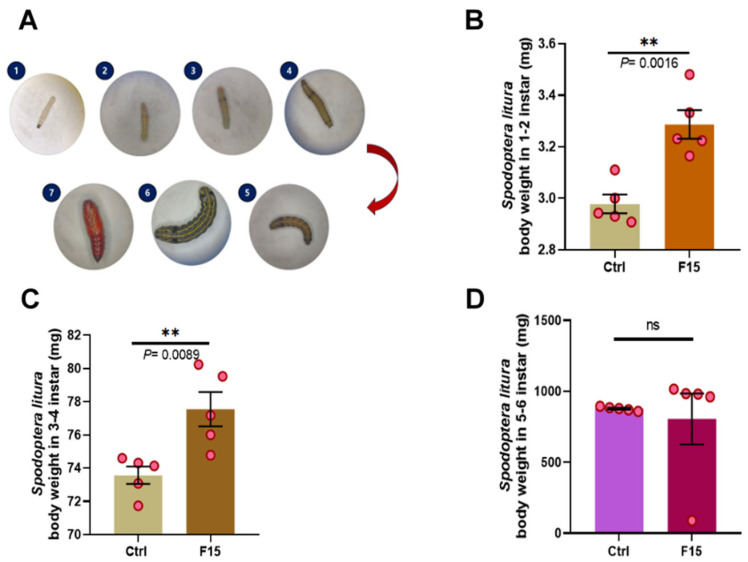
Developmental progression and body-weight responses of *Spodoptera litura* under control (CK) and formulation diet 15 (F15). (**A**) The life cycle of *S. litura* across successive developmental stages (1–7 instars). (**B**) Body-weight measurements of CK and the F15 diet across early stages (1–2 instars). (**C**) 3–4 instars, and (**D**) 5–6 instars. Each development stage has five groups (*n* = 50). Data were analysed using Student’s *t*-test, and results are presented as mean ± SEM with asterisks indicating significant differences relative to control (** *p* < 0.01 ns: no significant.) Pink dots represent individual biological replicates (*n* =5 per group).

Larval development proceeded successfully through all instars under both dietary treatments; however, distinct differences in growth dynamics emerged across developmental stages. During the early instars (1st–4th), larvae reared on the F15 diet exhibited significantly greater body mass compared with those fed the control (CK) diet (1st–2nd instars, *p* = 0.0016; 3rd–4th instars, *p* = 0.0089), indicating enhanced early-stage growth performance (Figure 2). In contrast, during the later instars (5th–6th), no significant differences in body mass were observed between the two dietary treatments, suggesting that the growth advantage associated with F15 was not sustained at advanced developmental stages. Despite this convergence, larvae fed the F15 diet consistently maintained a higher overall body mass trajectory across development.

Importantly, all individuals completed the full developmental cycle under both diets, with no observable differences in survival or progression through the final instars. Collectively, these results indicate that while the F15 formulation promotes early larval growth, it does not significantly alter late-stage biomass accumulation or developmental completion.

**Figure 3:**
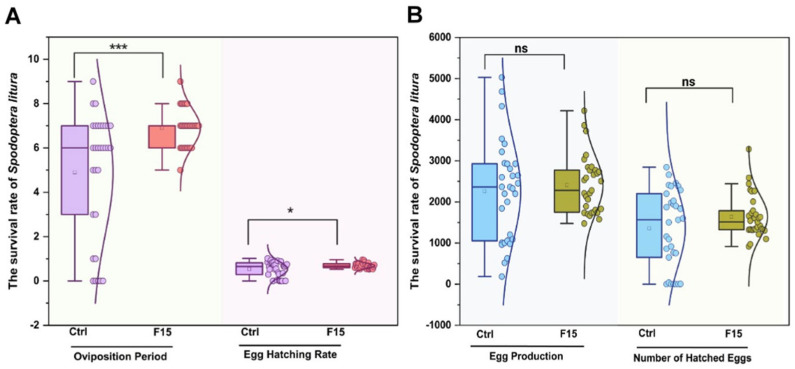
Dietary formulation of F15 enhances oviposition duration and egg viability. (**A**) The survival rate of the oviposition period and egg hatching rate in ctrl and F15. (**B**) The survival rate of egg production and number of hatched eggs in ctrl and F15. Data are presented as box-and-whisker plots (median, quartiles, and range) overlaid with violin plots depicting distribution density; individual biological replicates are shown as data points. Student’s *t*-tests are presented as mean ± SEM, indicating significant differences relative (* *p* < 0.05; *** *p* < 0.001, ns: no significant). Blue and orange dots represent individual biological replicates for the Ctrl and F15 groups, respectively (*n* = 5 per group).

To evaluate the effects of the F15 dietary formulation on reproductive performance, key oviposition parameters were systematically assessed. Females reared on the F15 diet exhibited a significantly prolonged oviposition period compared with CK individuals (Figure 3A, left panel, *p* = 0.0004), indicating sustained reproductive activity over time. Consistently, the egg hatching rate was also significantly higher in the F15 treatment group (Figure 3A, right panel, *p* = 0.0174), suggesting enhanced embryonic viability.

Despite these improvements, total reproductive output was not significantly altered. Although F15-fed females showed a numerical increase in total egg production relative to controls, this difference did not reach statistical significance (Figure 3B). Similarly, the total number of hatched offspring exhibited an increasing trend under F15 supplementation, but this effect was not statistically significant.

**Figure 4:**
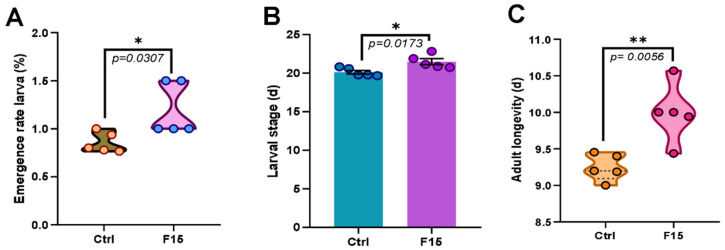
Dietary formulation F15 augments oocyte production and extends developmental duration and adult longevity. (**A**) Emergence rate of larvae in ctrl and F15 treatment. (**B**) Larval developmental stage in ctrl and F15-treated. (**C**) Adult longevity of ctrl and F15. Data are displayed with individual replicates overlaid on distribution plots showing median and variability. Blue and orange dots represent individual biological replicates for the Ctrl and F15 groups, respectively (*n* = 5 per group). Statistical analyses were performed using Student’s *t*-test, indicating significant differences relative (* *p* < 0.05; ** *p* < 0.01).

To further investigate the underlying basis of reproductive performance, oogenesis was examined. Quantitative analysis revealed that F15-fed females exhibited a significantly higher number of oocytes compared with control individuals (Figure 4A, *p* = 0.0307), indicating enhanced gametogenic capacity. In addition to reproductive traits, developmental timing and lifespan were assessed. Larvae reared on the F15 diet displayed a modest but statistically significant extension of the larval developmental period (Figure 4B, *p* = 0.0173). Notably, adult longevity was substantially increased in F15-treated individuals relative to the control group (Figure 4C, *p* = 0.0056).

Collectively, these results demonstrate that the F15 dietary formulation enhances reproductive potential by prolonging the oviposition period and improving egg viability, while also promoting increased oocyte production and extended lifespan.

### 3.2. Differential Metabolite Analysis and Identification

**Figure 5:**
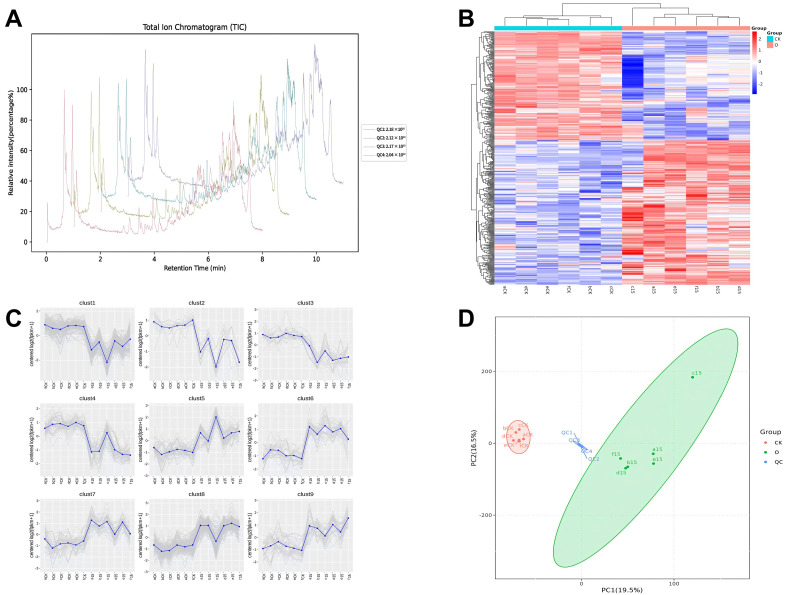
LC-MS metabolomic analysis reveals distinct metabolic signatures across experimental groups. (**A**) Total ion chromatograms (TIC) from representative samples showing metabolite elution profiles across experimental QC groups and four replications. (**B**) Hierarchical clustering heatmap of metabolite abundances across samples. (**C**) Metabolite cluster trend analysis for nine k-means clusters (clust1–clust9). Individual metabolite trajectories (grey) and mean cluster profiles (blue line with 95% confidence interval) across treatment groups. The y-axis shows centred log_2_ ratio-transformed abundance. (**D**) Principal component analysis (PCA) of metabolomic profiles separates treatment groups (CK, red; O, green; QC, blue). Ellipses represent 95% confidence intervals.

Metabolomic profiling revealed clear and consistent differences in chemical composition among the experimental groups. The LC–MS total ion chromatograms (TICs) displayed well-resolved retention-time patterns across 0–12 min, with most metabolites eluting between 1 and 8 min (Figure 5A). This distribution indicates efficient detection of diverse metabolite classes and stable instrument performance. Early-eluting compounds (RT < 3 min) and late-eluting metabolites (RT > 6 min) showed distinct abundance differences between treatments, suggesting diet-driven shifts across multiple chemical groups.

Hierarchical clustering of the full metabolite abundance matrix further demonstrated strong metabolic divergence among groups. The heatmap revealed two major sample clusters that corresponded closely to treatment identity. Samples from F15 O (red) accumulated higher levels of several metabolite sets, whereas CK (cyan) displayed a different, more uniform metabolic profile (Figure 5B). These contrasting clusters indicate that dietary formulation substantially remodels metabolic pathways, producing coordinated changes across broad chemical networks.

To better understand these response patterns, cluster-trend analysis was performed across nine k-means clusters (clust1–clust9), incorporating CK, O, and QC samples. Each cluster exhibited a distinct abundance trajectory; cluster 1 showed a gradual decrease from CK to O and QC, reflecting a consistent treatment-induced reduction. In contrast, clusters 3 and 6 displayed clear increases in the O group, suggesting activation or compensatory elevation of specific metabolic pathways. The remaining clusters (2, 4, 5, 7, 8, and 9) showed more complex, non-linear trends, highlighting the multifactorial nature of diet-responsive metabolic regulation (Figure 5C).

Principal component analysis further supported these findings. The first two principal components explained 40.1% of the total variance (PC1: 19.5%; PC2: 16.5%). Control samples (CK, red) were tightly grouped, indicating a stable and homogeneous metabolic state. QC samples (blue) occupied an intermediate position, demonstrating partial overlap with both groups. Notably, treatment samples (O, green) separated distinctly along the positive PC1 axis and exhibited wider dispersion along PC2 (Figure 5D). This separation indicates not only strong treatment effects but also increased metabolic diversity within the treated group. The clear spatial partitioning highlights PC1 as the major axis capturing diet-induced metabolic reprogramming.

Together, these multivariate analyses provide compelling evidence that dietary treatment drives substantial and coordinated metabolic restructuring. Distinct abundance patterns, cluster-specific trajectories, and strong ordination-space separation collectively demonstrate that the optimised diet induces broad biochemical shifts rather than isolated metabolite changes.

**Figure 6:**
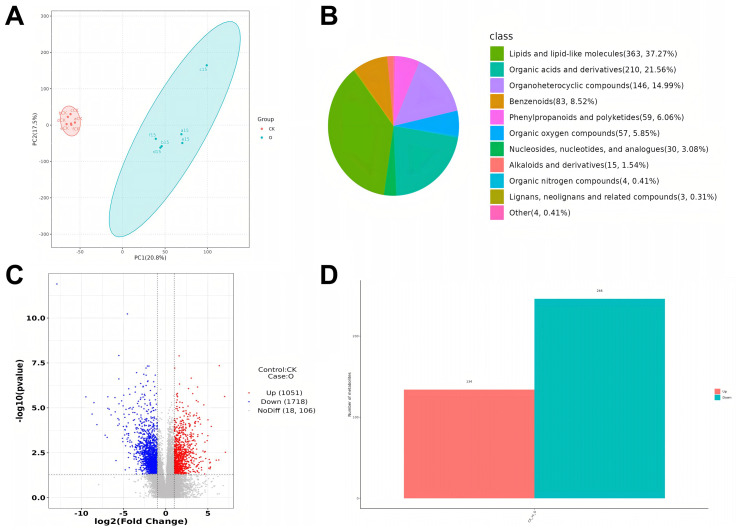
Differential metabolite analysis reveals treatment-induced metabolic reprogramming with class-specific enrichment. (**A**) Partial Least Squares Discriminant Analysis (PLS-DA) score plot discriminating control (CK, red) and F15 treatment (O, cyan) groups. (**B**) Pie chart showing compositional distribution of annotated metabolite classes. (**C**) Volcano plot depicting differential metabolite accumulation between treatment groups. X-axis shows log_2_ fold-change; the Y-axis shows −log_10_ *p*-value. Red points indicate significantly upregulated metabolites; blue points indicate downregulated metabolites (log_2_FC > 1, *p* < 0.05); grey points represent non-significant changes. (**D**) Bar chart comparing numbers of upregulated and downregulated metabolites between two groups (ctrl and F15).

Differential metabolite accumulation distinguishes treatment groups with class-specific enrichment patterns. Building upon the global metabolomic landscape, we next examined treatment-induced alterations in metabolite composition. PLS-DA successfully discriminated control (CK, red) from treatment (O, cyan) samples along the first component (Figure 6A), demonstrating robust metabolomic separation and validating treatment efficacy. The model’s discriminatory power was further confirmed through cross-validation, indicating reliable classification performance.

CK and O (treatment F15) samples resolved cleanly along the first component, confirming robust metabolic differentiation and validating the diet’s impact. Annotation of detected metabolites revealed that lipids and lipid-like molecules were the dominant chemical class (37.27%), followed by organic acids and derivatives (21.56%) and organoheterocyclic compounds (14.99%) (Figure 6B). The presence of diverse lipid subclasses including saturated, monounsaturated, and polyunsaturated fatty acids suggests extensive remodelling of lipid metabolism in response to dietary treatment.

Volcano plot analysis further identified metabolites that were significantly altered between CK and O groups (F15). Numerous features exceeded both fold-change and significance thresholds (log_2_FC > 1, *p* < 0.05). The number of upregulated metabolite features was 1051, downregulated was 1718, and no different was 18,106. This asymmetric distribution indicates that the optimised diet predominantly enhances biosynthetic activity rather than suppressing metabolic pathways.

Quantitatively, treatment group Ctrl versus O exhibited a markedly higher number of downregulated (246 metabolites) compared with upregulated features (234 metabolites) (Figure 6D). This nearly two-fold increase highlights strong anabolic metabolic responses induced by the diet, consistent with enhanced nutrient availability and increased metabolic flux across lipid, amino acid, and secondary metabolite pathways.

Collectively, these results demonstrate that the optimised diet triggers extensive metabolic reprogramming characterised by treatment-specific metabolite accumulation, lipid-class enrichment, and strong multivariate discrimination.

### 3.3. Functional Enrichment Analysis Reveals Coordinated Perturbation of Lipid Metabolism

**Figure 7:**
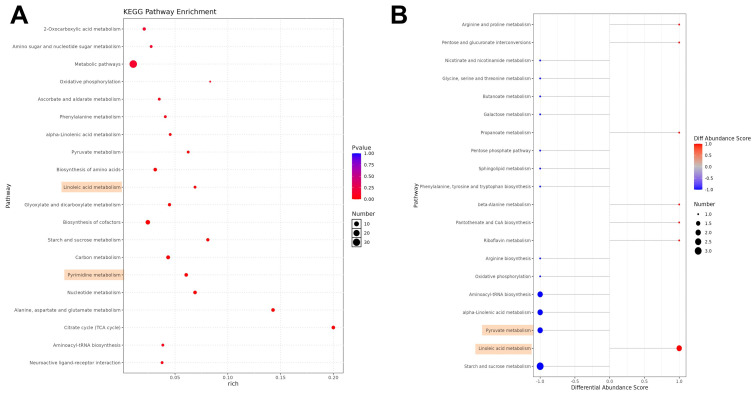
KEGG pathway enrichment analysis identifies lipid metabolism and amino acid biosynthesis as primary targets of metabolic reprogramming. (**A**) Bubble plot showing significantly enriched KEGG pathways. X-axis represents rich factor (ratio of differentially abundant metabolites to total pathway metabolites); the Y-axis lists pathway names. Bubble size indicates the number of enriched metabolites; colour represents −log_10_ (*p*-value), with purple indicating higher significance. (**B**) Differential abundance score (DAS). Bubble size indicates the different abundance scores; colour represents −log_10_ (*p*-value), with red indicating higher significance. The pathway names highlighted in yellow (e.g., pyruvate metabolism and α-linolenic acid metabolism) are specifically emphasized to indicate their prominent roles in the metabolic reprogramming.

To elucidate the biochemical pathways underlying the observed metabolite alterations, Kyoto Encyclopaedia of Genes and Genomes (KEGG) enrichment analysis was performed on differentially abundant metabolites. This analysis revealed significant enrichment across multiple metabolic pathways, with lipid-associated processes emerging as the most prominently affected category (Figure 7A). Notably, α-linolenic acid metabolism exhibited the highest level of enrichment, followed by linoleic acid metabolism and the biosynthesis of unsaturated fatty acids, indicating extensive remodelling of polyunsaturated fatty acid (PUFA) metabolism in response to the F15 diet.

Further examination of pathway-specific changes using differential abundance score (DAS) analysis revealed a clear directional shift in metabolite profiles (Figure 7B). Pathways related to linolenic acid metabolism contained the greatest number of altered metabolites, the majority of which were upregulated, suggesting enhanced PUFA-associated metabolic activity under the F15 treatment. Consistent upregulation patterns were also observed in linoleic acid metabolism and unsaturated fatty acid biosynthesis pathways. In contrast, several secondary metabolic pathways displayed mixed patterns of up- and downregulation, reflecting more complex and potentially compensatory metabolic adjustments.

Importantly, linoleic acid (18:2n-6), previously identified as a key discriminatory metabolite, was significantly enriched in F15-fed larvae, further distinguishing their metabolic profile from that of control individuals.

Collectively, these results demonstrate that the optimised F15 diet induces a coordinated metabolic reprogramming centred on lipid metabolism. The pronounced activation of PUFA-related pathways, together with the accumulation of linoleic acid, highlights the pivotal role of lipid metabolic processes in mediating the physiological and developmental responses to dietary intervention.

## 4. Discussion

### 4.1. Dietary Optimisation Enhances Developmental Performance Through Metabolic Reprogramming

Our results reveal that F15 dietary intervention significantly extended both larval developmental duration and adult lifespan compared to controls, while simultaneously improving oviposition period and egg hatching rates. This pattern of prolonged development coupled with enhanced reproductive output and longevity represents a departure from typical life history trade-offs. Recent metabolomic studies on *S. litura* fed various diets have similarly documented diet-dependent physiological differences, with corn-based formulations supporting superior growth metrics compared to natural plant diets [2] (Figure 2). However, our work extends these observations by demonstrating that metabolic architecture, particularly lipid composition, mechanistically underlies these phenotypic improvements.

The metabolomic profiles revealed coordinated upregulation of multiple biosynthetic pathways under F15 treatment, with lipid metabolism dominating the enrichment landscape. Specifically, alpha-linolenic acid metabolism, linoleic acid metabolism, and biosynthesis of unsaturated fatty acids exhibited the strongest enrichment signatures. Studies on silkworm (*Bombyx mori*) rearing demonstrate that artificial diets versus natural mulberry leaves elicit distinct haemolymph metabolic responses [23], emphasising that dietary composition profoundly influences insect metabolic homeostasis beyond simple nutritional adequacy. The predominance of lipid metabolic remodelling in F15-fed larvae suggests that this diet provides enhanced fatty acid precursors critical for membrane biosynthesis, energy storage, and signalling molecule production, all essential for the observed physiological improvements.

### 4.2. Linoleic Acid as a Longevity-Promoting Metabolite

Among the discriminatory metabolites identified in this study, linoleic acid exhibited the most pronounced elevation in F15-treated larvae. It is important to note, however, that the present data are correlational in nature; although linoleic acid levels showed a consistent positive association with improved longevity and selected reproductive traits, establishing a causal relationship would require direct dietary supplementation or genetic manipulation experiments, which fall outside the scope of the present study. Notwithstanding this limitation, the biological plausibility of a LA-mediated contribution to longevity is supported by a growing body of evidence. As an essential omega-6 polyunsaturated fatty acid, linoleic acid serves as a biosynthetic precursor for eicosanoids and endocannabinoids, bioactive lipid mediators that regulate immune function, energy homeostasis, and reproductive signalling in insects [24]. Recent investigations have further highlighted the anti-inflammatory, antifibrotic, and metabolic regulatory activities of specific long-chain fatty acids, with certain members of this class demonstrating longevity-promoting effects in model organisms at a mechanistic level broadly comparable to established compounds such as rapamycin and metformin [24]. The relevance of linoleic acid to insect lipid physiology is also supported by inter-species evidence: linoleic acid has been reported as a predominant fatty acid constituent of the lipid fraction in *Tenebrio molitor* larvae and *Acheta domesticus* [25,26], suggesting that elevated linoleic acid abundance may be a conserved feature of metabolic states associated with favourable growth and fitness outcomes across lepidopteran and coleopteran species.

Our metabolomic data support this interpretation, as F15-fed larvae exhibited elevated metabolite abundance consistent with enhanced biosynthetic capacity rather than catabolism-driven nutrient depletion. This suggests linoleic acid may optimise the balance between anabolic growth and catabolic maintenance, a fundamental requirement for extended lifespan. Beyond the structural and energetic roles discussed above, the marked elevation of linoleic acid in F15-fed larvae warrants particular attention from a functional perspective. Linoleic acid is a precursor for the biosynthesis of eicosanoids, a class of bioactive lipid mediators that regulate diverse physiological processes in insects, including immunity, reproduction, and stress responses [27]. Eicosanoids are known to mediate hemocyte motility, phagocytosis, encapsulation, and melanisation—key components of the insect innate immune system [28]. Therefore, the LA-enriched metabolic profile observed in F15-fed larvae raises the intriguing possibility that these insects may exhibit altered immune competence and, consequently, differential susceptibility to entomopathogens (bacteria, fungi, or viruses) or chemical insecticides. It is also plausible that the improved host quality associated with the F15 diet could enhance the performance of natural enemies, such as the egg parasitoid *T. remus*, which is widely used in biological control programs against *S. litura* [12,13]. We emphasise, however, that these remain correlative inferences; direct experimental validation through immune functional assays, pathogen challenge tests, and parasitoid bioassays is required to establish causal relationships. Nonetheless, our metabolomic data provide a strong mechanistic foundation and clear testable hypotheses for such future investigations. Besides the diet, individual species and environmental conditions, the stage of life can also influence the fatty acid profile of insects. Insects can biosynthesize/accumulate different fatty acids at various stages of life, depending upon the utility of fatty acids in the body [29]. Moreover, some studies have shown that oleic acid and linoleic acid treatments improved the conidial germination rate on water agar plates and locust hindwings and increased the growth and development of *M. rileyi* [30], suggesting that exogenous oleic acid and linoleic acid provide energy substances for *M. rileyi*’s growth and development [31]. Among the lipids, linoleic acid significantly promoted the growth and development of *M. rileyi* and enhanced its stress tolerance and virulence [32].

By increasing the proportion of saturated fatty acids like linoleic acid while maintaining adequate polyunsaturated fatty acids (PUFAs) for essential functions, the F15 diet may confer optimal membrane resilience against oxidative damage. Ferroptosis results from failure of lipid quality control systems when PUFA-containing membrane phospholipids undergo excessive peroxidation [33,34], a process that accelerates during ageing and metabolic stress. The elevated linoleic acid in F15-fed larvae may thus provide structural membrane protection while supporting mitochondrial integrity, collectively contributing to extended adult longevity.

### 4.3. Dietary Lipid Composition and Metabolic Health

Beyond linoleic acid, our pathway enrichment analyses revealed coordinated upregulation of multiple fatty acid metabolic pathways, including palmitic acid, stearic acid, linoleic acid, and alpha-linolenic acid metabolism. This metabolic signature indicates that F15 provides a balanced lipid profile supporting both saturated and unsaturated fatty acid synthesis (Figure 7). Protein and carbohydrate levels in artificial diets are fundamental determinants of lepidopteran growth and development, influencing developmental period, body weight, pupation rate, and adult emergence [35]. However, our findings emphasise that lipid quality—not merely quantity constitutes an equally critical nutritional dimension.

The predominance of upregulated versus downregulated metabolites (1051 vs. 1718 features) suggests F15 promotes predominantly anabolic metabolic states (Figure 6D). This pattern aligns with the observed improvements in body weight during early instars and enhanced reproductive capacity. Recent meta-omics investigations of *S. litura* reveal that artificial diets profoundly alter gut microbiota-metabolome interactions, with microbe-metabolite crosstalk differing markedly between natural plant-fed hosts and those consuming artificial formulations [36]. These microbial-metabolic interactions may amplify or modulate the direct nutritional effects of F15, warranting future investigation into how diet-microbiome-host metabolism networks jointly influence longevity outcomes.

### 4.4. Implications for Mass-Rearing and Pest Management

From an applied perspective, our findings demonstrate clear advantages of the F15 corn-based diet for *S. litura* mass-rearing. The extended oviposition period, elevated egg hatching rates, and increased adult longevity collectively enhance production efficiency for laboratory colonies. Furthermore, the significantly higher emergence rate (1.20 ± 0.27% vs. 0.85 ± 0.10%, *p* = 0.0173) and reduced variability in several life-history parameters suggest that the F15 diet may improve the consistency and reproducibility of laboratory-reared insects, thereby generating a more reliable experimental model for studies requiring standardised insect material. However, we acknowledge that this inference is based on single-generation data; formal multi-generational stability tests are needed to confirm the long-term reliability of the F15 diet.

Mass-rearing of *S. litura* is essential for implementing various pest control strategies, including biological control agent production and sterile insect technique programs [2]. Improved diet formulations that enhance insect quality and reproductive output directly translate to more cost-effective and sustainable rearing operations. In particular, the production of high-quality hosts is a critical determinant of parasitoid fitness in biological control programs. For example, *T. remus*, an egg parasitoid used against noctuid pests, maintains stable parasitism rates when reared on *S. litura* eggs [12,13]. The improved host quality associated with the F15 diet—evidenced by enhanced oocyte production, prolonged oviposition, and higher egg viability—may therefore translate to improved parasitoid performance, a hypothesis that warrants direct experimental testing in future studies.

### 4.5. Addressing Limitations and Future Directions

Several important limitations of the present study should be acknowledged. First, our metabolomic profiling provides correlative rather than causal evidence. While we observed consistent associations between linoleic acid enrichment and improved phenotypic traits, direct supplementation experiments or genetic manipulations (e.g., knockdown of fatty acid desaturases) are required to establish causality. Second, we did not assess the impact of the F15 diet on insect immune function, pathogen resistance, or insecticide susceptibility. Although our data offer a strong mechanistic rationale—particularly through the LA-eicosanoid axis—these hypotheses remain to be experimentally validated. Third, the long-term stability and reproducibility of the F15 diet across multiple generations were not evaluated, which is a critical prerequisite for recommending its adoption in large-scale rearing facilities. Fourth, our metabolomic analysis focused exclusively on gut tissue; extending this approach to haemolymph, fat body, and reproductive organs would provide a more comprehensive view of systemic metabolic reprogramming. Finally, the role of gut microbiota in mediating or modulating diet-induced metabolic shifts warrants dedicated investigation, given the well-documented influence of microbial communities on host lipid metabolism and fitness in lepidopteran species [32].

Despite these limitations, our study provides a valuable theoretical framework and a clear set of testable hypotheses for future research. In particular, the identification of linoleic acid as a key discriminatory metabolite offers a concrete molecular target for optimising artificial diets not only for *S. litura* but also for other insect species reared under laboratory or industrial conditions. We anticipate that the F15 diet, with its demonstrated advantages in growth, reproduction, and longevity, will serve as a robust platform for both fundamental studies in insect nutritional physiology and applied efforts in sustainable pest management.

## 5. Conclusions

This study demonstrates that a nutritionally optimised corn-based artificial diet (F15) induces coordinated metabolic reprogramming in *S. litura*, with linoleic acid emerging as a key metabolite associated with enhanced growth, reproductive performance, and extended adult longevity. Untargeted metabolomic analysis revealed a consistent enrichment of lipid metabolic pathways, particularly those involved in polyunsaturated fatty acid biosynthesis, highlighting the central role of lipid metabolism in mediating diet-induced physiological responses.

These findings provide mechanistic insight into how dietary composition shapes insect development and life-history traits, while underscoring the nutritional and functional advantages of corn-based formulations for large-scale rearing systems. The identification of linoleic acid as a critical metabolic mediator offers a valuable framework for rational diet optimisation across insect species.

Several important directions remain open for future investigation. The present study focused exclusively on gut metabolomics; extending this approach to haemolymph, fat body, and reproductive tissues would provide a more comprehensive picture of how dietary fatty acid enrichment reshapes systemic metabolic networks, including neuroendocrine and immune signalling pathways that may jointly mediate the reproductive and longevity outcomes observed here. The role of gut microbiota in mediating or modulating diet-induced metabolic shifts also warrants dedicated investigation, given the well-documented influence of microbial communities on host lipid metabolism and fitness in lepidopteran species. From a mechanistic standpoint, the metabolic associations and hypothesised pathways proposed in this study, particularly the LA-AA-endocannabinoid axis, require functional confirmation through dietary LA supplementation experiments and targeted gene expression analyses, including qPCR quantification of key enzymes such as FADS2 and MAGL. Finally, the scalability and cost-effectiveness of LA-enriched corn-based diets under industrial rearing conditions remain to be evaluated across multiple generations, which will be essential before these nutritional strategies can be recommended for large-scale *S. litura* mass-rearing programmes.

Importantly, while our findings establish correlative links between dietary lipid composition, metabolic reprogramming, and phenotypic improvements, they do not provide direct evidence for altered insecticide susceptibility, pathogen resistance, or multi-generational stability. We have explicitly discussed these as limitations and outlined specific experimental approaches—including direct LA supplementation, immune functional assays, and multi-generational rearing studies—to address them in future work. By doing so, we hope to provide not only a practical diet formulation but also a mechanistic framework that guides subsequent research into the nutritional regulation of insect health, immunity, and fitness.

## Figures and Tables

**Table 1:** Artificial diet formulations.

Diet Formula	Maize Powder (g)	Soybean Powder (g)	Yeast Powder (g)	Wheat Bran (g)	Cost	Diet Formula	Maize Powder (g)	Soybean Powder (g)	Yeast Powder (g)	Wheat Bran (g)	Cost
CK	0	100	40	60	42.32	9	60	40	40	60	39.8
1	0	100	20	80	36.68	10	60	40	20	80	35.15
2	0	100	0	100	31.04	11	60	40	0	100	30.04
3	20	80	40	60	41.82	12	80	20	40	60	40.32
4	20	80	20	80	36.18	13	80	20	20	80	34.68
5	20	80	0	100	30.54	14	80	20	0	100	29.04
6	40	60	40	60	41.32	15	100	0	40	60	39.82
7	40	60	20	80	35.68	16	100	0	20	80	34.18
8	40	60	0	100	30.04	17	100	0	0	100	28.54

Note: The composition of vitamins and preservatives was as follows: vitamin C (4 g), niptibet (2 g), orbic acid (2 g), cholesterol (0.8 g).

## Data Availability

The original contributions presented in this study are included in the article. Further inquiries can be directed to the corresponding authors.
